# Hepatitis B virus infection and associated factors among pregnant women attending antenatal clinics in West Hararghe public hospitals, Oromia region, Ethiopia

**DOI:** 10.11604/pamj.2020.35.128.17645

**Published:** 2020-04-18

**Authors:** Belay Mamuye, Tesfaye Gobena, Lemessa Oljira

**Affiliations:** 1 Ethiopian Field Epidemiology and Laboratory Training Program (EFELTP), Haramaya University, Harar, Ethiopia; 2 College of Health and Medical Science, Haramaya University, Harar, Ethiopia

**Keywords:** Hepatitis B virus, seroprevalence, hepatitis B surface antigen

## Abstract

**Introduction:**

Globally, approximately 350-400 million persons are chronically infected with hepatitis B virus (HBV), over 65 million of whom are in Africa. One in four people with chronic hepatitis B develop serious health problems. Mother-to-child transmission (MTCT) is responsible for more than half of chronic infections. If infected at birth, a child has a 90% chance of becoming a chronic carrier. We evaluated hepatitis B virus prevalence and risk factors for infection among pregnant women attending antenatal clinics in West Hararghe public hospitals, Oromia region, Ethiopia.

**Methods:**

We conducted a cross-sectional study among 363 pregnant women at routine antenatal clinic visits in West Hararghe public hospitals from April-May, 2017. We used systematic random sampling method to enroll participants. We used a structured questionnaire to collect information on risk factors, and collected blood samples to test for hepatitis B Virus surface antigen (HBsAg) by enzyme-linked immunosorbent assay (ELISA). Data were entered using EpiData Version.3.1 and exported to SPSS Version 23.0 for descriptive analyses and binary logistic regression

**Results:**

The overall seroprevalence of HBsAg among participants was 6.1% (95% CI 3.9-8.5). History of abortion (aOR=4.3, 95% CI 1.3-15.0), traditional tonsillectomy (tonsillectomy conducted by an untrained practitioner) (aOR=4.4, 95% CI 1.1-17.8), admission to a health facility (aOR=4.4, 95% CI 1.2-16.9), multiple sexual partners (aOR=6.3, 95% CI 1.7-23.4) and familial liver disease (aOR=8.2, 95% CI 2.1-32.8) were associated with hepatitis B virus infection among pregnant women.

**Conclusion:**

The prevalence of hepatitis B virus in study area indicates a high-intermediate level epidemic. Multiple types of healthcare, as well classic risk factors such as multiple sex partners and a family history of liver disease increased the odds of infection. Hygiene promotion and infection prevention methods in healthcare settings are recommended to avoid nosocomial infections. To reduce MTCT, we recommended screening all pregnant women for hepatitis B virus as part of routine antenatal care and supportive treatment and making available methods of preventing infection at birth, including prophylaxis and birth dose vaccine.

## Introduction

Hepatitis B is a potentially life-threatening liver infection caused by the hepatitis B virus (HBV) [1]. Chronically infected persons have an increased lifetime risk for cirrhosis and hepatocellular carcinoma (HCC). Worldwide, about 2 billion persons have been infected with HBV; an estimated 350-400 million are chronically infected, of whom at least 65 million are in Africa. More than 800,000 people die every year due to complications of HBV infection [1-3]. Mother-to-child transmission (MTCT) accounts for more than half of chronic HBV infection worldwide, despite an existing immunoprophylaxis regimen [4]. Children born to hepatitis B surface antigen-positive (HBsAg+) and hepatitis B envelope antigen-positive (HBeAg+) mothers have a 70-90% chance of prenatal acquisition of HBV infection and 85-90% of them will become chronic carriers of the disease. Beyond this, viral hepatitis during pregnancy is associated with a high risk of maternal complications and maternal mortality. As a result, the World Health Organization recommends screening pregnant women for infection, providing monovalent HBV vaccination and hepatitis B immune globulin (HBIg) to neonates within 24 hours of birth and decreasing viral replication with antiretroviral therapy for pregnant women [1, 5]. Previous studies in Ethiopia have indicated an HBV prevalence among pregnant women of 3.8% to 7.8% [6, 7]. However, HBV screening during pregnancy is not practiced routinely in health facilities in Ethiopia. Evidence-based information on factors associated with HBV infections in pregnant women are needed to promote policy changes to reduce maternal infections and prevent MTCT of HBV. We aimed to determine seroprevalence of hepatitis B infection and its associated risk factors among pregnant women attending antenatal clinics in West Hararghe Zone public hospitals, Oromia region, Ethiopia.

## Methods

**Study design:** we conducted a cross-sectional study in West Hararghe Zone, located in Eastern Oromia region, Ethiopia, from April-May 2017. According to the projection for 2017 based on the 2007 census, the total population of the zone is approximately 2.4 million persons. There are 2 public hospitals, 80 health centers and 449 health posts in the zone during the study period (Source: West Hararghe Zonal health office). Eligible participants were pregnant women attending antenatal care clinics in West Hararghe public hospitals.

**Sample size determination and sampling methods:** sample size was calculated for prevalence using a single population proportion formula. We estimated sample size based on a 7.8% prevalence of HBV infection [7], a precision of 4%, 95% confidence and a 5% non-response rate. Using these assumptions, the calculated sample size was 363 participants. We estimated that 1,278 pregnant women would receive routine antenatal care at antenatal care (ANC) clinics in two hospitals in West Hararghe Zone during the two months of the study (480 pregnant women at Chiro Zonal Hospital and 798 pregnant women at Gelemso General Hospital). Participants were selected proportionally from each hospital using systematic random sampling. Accordingly, 136 and 227 pregnant women attending antenatal clinics were selected from Chiro and Gelemso general hospital ANCs clinics respectively. To avoid repetition, an identification mark was included on the participant's clinical card and the unique medical record number (MRN) was used for study registration.

### Data collection methods

**Face-to-face interview:** we used a pretested, structured questionnaire adapted from the WHO protocol for assessment of hepatitis B virus infection in antenatal care clinics [8] to collect information. Exposures of interest included socio-demographics, medical and health-related factors, health behaviors and practices (circumcision, tattooing, ear and nose piercing, traditional tonsillectomy (complete or partial removal of tonsils by informal practitioner at home), risky sexual behavior, history of abortion (spontaneous or deliberate), history of ever being admitted to a health facility) and other characteristics. Questionnaires were prepared in English and translated to the local language (Oromiffaa), pretested on 30 pregnant women at ANC clinics, and administered by four trained, diploma-level midwives through face-to-face interviews. All participants gave written consent.

**Laboratory investigation of HBsAg:** after interview, approximately 4ml of venous blood was collected with EDTA and SST tubes from each consented study participant. Serum was separated from whole blood cells, transferred to cryo-tubes and stored at -20°C before transport to a blood bank center. Serum was tested for HBsAg by ELISA using ADVANCED^®^ Diagnostic kit for HBV according to manufacturer's standard operating procedure (SOPs) [9]. Positive and negative controls were included to verify assay performance. Positive samples were re-tested to confirm the result.

**Data processing and analysis:** we coded and double-entered data using EpiData Version 3.1 and exported to SPSS version 23 for cleaning and further analysis. We performed descriptive analyses and bivariate logistic regression analysis to determine factors associated with HBV infection. Crude odds ratio (COR), p-values and 95% confidence intervals were calculated to investigate the association between each predictor variable and dependent variable. Variables with p<0.25 in bivariate analysis were entered into a multivariate model. Standard errors were used to detect multicollinearity.

**Ethical consideration:** ethical clearance was obtained from Haramaya University, Institutional Health Research Ethics Review Committee (IHRERC). All participants provided informed consent. Individuals testing positive for HBsAg were linked to the physician in the hospitals for appropriate counseling, case management and follow up. During the study period, birth dose monovalent HBV vaccine was started in the hospitals as a pilot program. All infants born to infected mothers were targeted to receive the vaccine.

## Results

**Socio-demographic characteristics of the study participants:** a total of 363 pregnant women were included in the study. The mean age of the participants was 23.7 years (standard deviation, 5.1 years; range 15-46 years). More than half of the participants (56.2%) were rural residents. Most (99.4%) were married and 21(5.8%) of these were polygamous. One hundred and forty-four (39.7%) participants had no formal education and 74 (20.4%) were housewives (Table 1).

**Table 1:** Socio-demographic characteristics of pregnant women

Characteristics		Frequency	Percentage (%)
Residence	Urban	159	43.8
	Rural	204	56.2
Age (in years)	15-24	191	52.6
	25-34 years	152	41.9
	35-49 years	20	5.5
Marital Status	Single	2	0.6
	Monogamous	340	93.6
	Polygamous	20	5.5
	Separated/divorced	1	0.3
Ethnicity	Oromo	315	86.8
	Amhara	37	10.2
	Other	11	3.0
Educational status	No formal education	144	39.7
	Primary School	145	39.9
	Secondary School	36	9.9
	Diploma and above	38	10.5
Occupation	Government employee	37	10.2
	Private	11	3
	Farmer	181	49.9
	Housewives	74	20.4
	Others	60	16.5
Gravidity	Primigravidae	110	30.3
	Multigravidae	253	69.7

**Prevalence of hepatitis B infection:** overall, 22 (6.1%; 95%CI=3.9%-8.5%) participants were seropositive for HBsAg. Of 137 participants at Chiro Zonal Hospital, 7 (5.1%) were positive; of 226 from Gelemso General Hospital, 15 (6.6%) were positive. Among 20 persons aged 35-40 years, 3 (15%) were positive. The prevalence of HBV infection was higher among polygamous pregnant women (30%) than monogamous pregnant women (4.7%). The prevalence of HBV decreased with educational level (Table 2).

**Table 2:** Prevalence of HBV among pregnant women

Variables		HBsAg Status		Total n (%)
		Positive n (%)	Negative n (%)	
Age (in years)	15-24	6(3.1%)	185(96.9%)	191(52.6%)
	25-34 years	13(8.6%)	139(91.4%)	152(41.9%)
	35-49 years	3(15.0%)	17(85.0%)	20(5.5%)
Residence	Urban	13(8.2%)	146(91.8%)	159(43.8%)
	Rural	9(4.4%)	195(95.6%)	204(56.2%)
Marital status	Single	0(0.0%)	1(100%)	1(0.3%)
	Monogamous	16(4.7%)	325(95.3%)	341(93.9%)
	Polygamous	6(30%)	(4.1%)	20(70%)
	Separated/divorced	0(0.0%)	1(100%)	1(0.3%)
Ethnicity	Oromo	18(5.7%)	297(94.3%)	315(86.8%)
	Amhara	2(5.4%)	35(9.6%)	37(10.2%)
	Somali	1(25%)	3(75%)	4(1.1%)
	Guraghe	1(14.4%)	6(8.6%)	7(1.9%)
Educational status	No formal education	13(9.0%)	131(91%)	144(39.7%)
	Primary school	7(4.8%)	138(95.2%)	145(39.9%)
	Secondary School	1(2.8%)	35(97.7%)	36(9.9%)
	Diploma and Above	1(2.6%)	37(97.4%)	38(10. %)
Participant Work	Government employee	1(2.7%)	36(97.3%)	37(10.2%)
	Private	2(18.2%)	9(81.8%)	11(3.0%)
	Farmer	14(7.7%)	167(92.3)	181(49.9%)
	Housewives	3(4.1%)	71(95.9%)	74(20.4%)
	Others	2(3.3%)	58(96.7%)	60(16.5%)
Gravidity	Primigravida	5(4.5%)	105(95.5%)	110(30.3%)
	Multigravida	17(6.7%)	236(9.3%)	253(69.7%)
Parity	Nullipara	6(4.0%)	144(96.0%)	150(41.3%)
	Primipara	4(5.8%)	65(94.2%)	69(19.0%)
	Multipara	12(8.3%)	132(91.7%)	144(39.7%)
Gestational age	First trimester	3(8.3%)	33(91.7%)	36(9.9%)
	Second trimester	11(6.3)	163(93.7%)	174(47.9%)
	Third trimester	8(5.2%)	145(94.8%)	153(42.1%)

**Factors associated with hepatitis B virus infection:** in bivariate analysis, age category, a history of home delivery by traditional birth attendants, a history of abortion, body tattooing, traditional tonsillectomy, admission to a health facility, having dental extraction, multiple sexual partners, and familiar history of liver disease were significantly associated with HBsAg positivity (Table 3). In multivariate analysis, history of abortion (AOR=4.3; 95%CI=1.3-15.0), traditional tonsillectomy (AOR=4.4; 95%CI=1.1-17.8), history of admission to health facility (AOR=4.4; 95%CI=1.2-16.9), multiple sexual partners (AOR=6.3; 95%CI=1.7-23.4) and history of liver diseases among family members (AOR=8.2; 95%CI=2.1-32.8) remained significantly associated with infection (Table 3). Residence of the participant, age category, blood transfusion, home delivery, female circumcision, tattooing, ear piercing and history of dental procedure had not shown significant association with HBV infection.

**Table 3:** Factors associated with HBV infection among pregnant women

Variables	HBsAg status		COR(95% C.I)	AOR(95% C.I)
	Positive	Negative		
**Residence**				^+^
Urban	13(8.2%)	146(91.8%)	1.93(0.80-4.64)	2.6(0.76-9.2)
Rural	9(4.4%)	195(95.6%)	1	
**Age in years**				
15-24	6(3.1%)	185(96.9%)	1	
25-34 years	13(8.6%)	139(91.4%)	2.9(1.1-7.8)	0.7(0.2-2.8)
35-49 years	3(15.0%)	17(85.0%)	5.4(1.3-23.7)	1.4(0.1-15.1)
**Home delivery by TBA**				^+^
No	7(3.6%)	189(96.4%)	1	
Yes	15(9%)	152(91%)	2.7(1.1-6.7)	1.8(0.4-6.9)
**History of abortion**				^+^
No	11(3.5%)	302(96.5%)	1	
Yes	11(22.0%)	39(78.0%)	7.7(3.2-19.0)	4.3(1.3-15.0) ^++^
**History of circumcision**				^+^
No	1(1.2%)	82(98.8%)	1	
Yes	21(7.5%)	259(92.5%)	6.7(0.9-50.2)	7.9(0.8-82.6)
**Body tattooing**				^+^
No	16(4.7%)	326(95.3%)	1	
Yes	6(28.6%)	15(71.4%)	8.2(2.8-23.8)	1.7(0.3-10.4)
**Traditional Tonsillectomy**				^+^
No	11(3.5%)	307(96.5%)	1	
Yes	11(24.4%)	34(75.6%)	9.0(3.6-22.4)	4.4(1.1-17.8) ^++^
**Ear Piercing**				
No	1(2.8%)	35(97.2%)	1	
Yes	21(6.4%)	306(93.6%)	2.4(0.3-18.4)	
**History of admission to health facilities**				^+^
No	12(3.8%)	301(96.2%)	1	
Yes	10(20.0%)	40(80.0%)	6.3(2.6-15.5)	4.4(1.2-16.9) ^++^
**History of dental procedure**				^+^
No	18(5.2%)	331(94.8%)	1	
Yes	4(28.6%)	10(61.4%)	7.4(2.1-25.8)	2.6(0.3-24.0)
**History of CS**				^+^
No	20(5.6%)	334(94.4%)	1	
Yes	2(22.2%)	7(77.8%)	4.77(0.9-24.5)	2.13(0.2-25.5)
**Blood Transfusion**				
No	21(5.9%)	337(94.1%)	1	
Yes	1(20%)	4(80%)	4.0(0.4-37.5)	
**Multiple sexual partners**				^+^
No	14(4.2%)	316(95.8%)	1	
Yes	8(24.2%)	25(75.8%)	7.2(2.8-18.9)	6.3(1.7-23.4) ^++^
**History of liver diseases among family members**				^+^
No	13(3.9%)	323(96.1%)	1	
Yes	9(33.3%)	18(66.7%)	12.4(4.7-32.9)	8.2(2.1-32.8) ^++^

1= reference category

+ = P value greater than 0.25 in bivariate analysis

++ = Significant variables in multivariate analysis at P<0.05

COR= Crude Odd Ratio, AOR= Adjusted Odd Ratio

## Discussion

We investigated the burden and risk factors for hepatitis B virus infection among pregnant women in Eastern Oromia region, Ethiopia. We found the overall prevalence to be 6.1%, varying slightly by hospital. Having a history of abortion, traditional tonsillectomy, admission to a health facility, multiple sexual partners and liver disease among family members were significantly associated with infection. According to the WHO, HBV endemicity can be classified as low (prevalence <2%), low-intermediate (prevalence 2-4.9%), high-intermediate (prevalence 5-7.99%, and highly endemic (≥8%) [10]. The HBV prevalence identified in this study is similar to that found in previous studies in Ethiopia, including in Debre Tabor Hospital, North West Ethiopia (5.3%) [11], Bahir Dar City, North Ethiopia (6.6%) [12] and Hawasa University Referral Hospital, Southern Ethiopia (7.8%) [7]. When compared with other African countries, results are also similar to the prevalence in Sudan (5.6%) [13] and Cameroon (7.7%) [14], but lower than other study conducted in Nigeria 11% [15], Mali (8.0%) [16], and Uganda (11.8%) [17]. Given the wide range of risk factors for HBV infection, these variations could be due to differences in cultural practices, sexual behaviors, sampling method and/or laboratory test methods employed to detect HBsAg.

Multiple risk factors in our study were associated with receipt of health care, both traditional (delivered by an untrained practitioner at home) and formal (delivered by a trained practitioner at a health facility). Procedures including removal of the uvula and/or tonsils are frequently practiced in parts of Ethiopia, including West Hararghe zone, outside the context of a formal healthcare facility [18]; approximately 1/3 of Ethiopians undergo traditional tonsillectomies during childhood [19]. A single surgical kit may be used on more than one child during these procedures which may increase the risk of HBV infection [20]. As abortion is only legal in cases of rape, incest, or to save the life of the mother in Ethiopia, induced abortions may also be performed in informal circumstances, increasing infection risk [21]. Poor hygiene, contaminated instruments and lack of personal protective equipment can put women at risk of multiple infections [22] during these procedures. Abortion is also frequently associated with unwanted and unsafe sexual intercourse, which may itself increase the risk of HBV infection [23]. Previous studies conducted among pregnant women in Ethiopia showed that abortion [22], but not traditional tonsillectomies [7, 24], were associated with HBV infection. In our study, both traditional tonsillectomy and abortion were associated with infection. It was not clear from our study whether abortions were induced or spontaneous, or whether they caused or were a result of HBV infection.

Beyond the risk conferred by traditional health procedures, infections may be acquired in formal healthcare settings. History of admission to a health facility was associated with HBV infection among pregnant women. This has also been found in other studies [20, 24]. In formal healthcare settings, patients are most often put at risk of HBV infection through exposure to body fluids [25, 26]. However, a study conducted in Addis Ababa did not find this to be a significant exposure [27]. The discrepancy may be due to the difference in hygiene and aseptic precautions regarding materials used in the procedures among the different health institution. As noted previously in multiple studies, having a history of multiple sexual partners [3, 6, 28, 29] and having a family history of liver disease [22, 30, 31] were both associated with HBV infection. As a sexually transmitted disease, the risk of HBV infection increases with the duration of sexual activity and number of sexual partners [6]. A family history of liver disease suggests that the woman may be a chronic carrier of the virus, having been infected by her family, possibly through mother-to-child transmission previously. However, blood transfusion [6, 22], history of dental procedure [32], female circumcision [13, 33], residence, tattooing and ear piercing [6, 7] all have failed to show any evidence of association with HB virus transmission in line with our current study. This may be explained by variations in study area and period, sample size and safety precautions being taken.

Limitations of our study included being unable to perform laboratory tests for serological markers of hepatitis B infection that could identify the infection stage and a lack of data on details. Despite these limitations, the study can provide evidence for policymakers to consider alternative strategies to decrease MTCT of HBV, prevent HBV infection and eliminate HBV infection in the country.

## Conclusion

The prevalence of HBsAg among pregnant mothers attending ANC clinics in West Hararghe public hospitals was high-intermediate. History of abortion, traditional tonsillectomy, history of admission to health facility, having multiple sexual partners and history of liver diseases among family members were independent factors associated with hepatitis B virus infection among pregnant women. All pregnant women should be screened for HBV as a part of routine antenatal care in ANC clinics and should be treated if positive. Avail birth dose HBV vaccine with HBIG and strengthening routine immunization was recommended to prevent MTCT of HBV. Also, promotion of hygiene and infection prevention methods in healthcare setting is recommended order to avoid nosocomial infections. Moreover, household members and sexual partners of HBV carriers should need to be identified through prenatal screening (contact tracing) and if susceptible, should receive HBV vaccine and awareness should be given for family members on HBV infection prevention.

### What is known about this topic

- Africa and Asia have the highest prevalence of HBV worldwide, with >8% HBsAg positivity;
- Risk factors of HBV infection in pregnant women varies among communities depending on their culture and practices.

### What this study adds

- The prevalence of HBsAg among pregnant mothers attending ANC clinics in West Hararghe Public hospitals was found to be 6.1%;
- History of abortion, traditional tonsillectomy, history of admission to health facility, having multiple sexual partners and history of liver diseases among family members were identified as factors associated with HBV infection;
- An evidence to expand HBV screening of all pregnant women in ANC clinics and to start birth dose HBV vaccine.

## Competing interests

The authors declare no competing interests.
